# Supplementary material for: Deep photonic network platform enabling arbitrary and broadband optical functionality
Source: Nat Commun. 2024 Feb 16;15:1432. doi: 10.1038/s41467-024-45846-3 (PMC10873373; doi:10.1038/s41467-024-45846-3)
Supplement: Supplementary file 1 — Supplementary Information [file 41467_2024_45846_MOESM1_ESM.pdf]

Deep Photonic Network Platform Enabling Arbitrary and Broadband Optical Functionality: Supplemental Information

Ali Najjar Amiri[1], Aycan Deniz Vit[1], Kazim Gorgulu[1], Emir Salih Magden[1,*]

[1]*Department of Electrical and Electronics Engineering,*

Koç University, Sariyer, Istanbul, 34450, Turkey

**Corresponding author: esmagden@ku.edu.tr*

I. MODELING OF DIRECTIONAL COUPLERS IN MZI INTERFEROMETERS

For physical accuracy of the transfer matrix model employed, the complete optical transmission including any losses through the S-bends and directional couplers are incorporated in $T(\lambda)$, by using the optical response of the directional coupler obtained from 3D-FDTD simulations, as shown in Supp. Fig. 1.

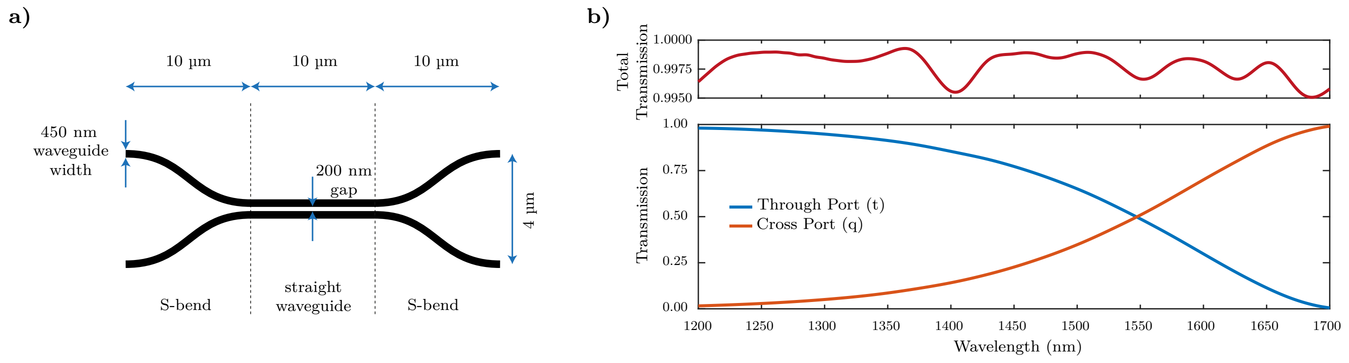

Supplementary Figure 1. Diagram and optical response of the directional coupler. (a) Diagram of the directional coupler indicating important geometrical parameters including 10 µm-long straight coupling section with 200 nm gap, 10 µm-long S-bends, 4 µm center-to-center waveguide separation between the top and the bottom arm at the start and end of the directional coupler, and 450 nm waveguide width through the entire directional coupler. **(b)** Optical responses at the cross- and through-port of the directional coupler used in our deep photonic networks. Results were obtained from 3D-FDTD simulations performed with a maximum spatial discretization of 17 nm in all three dimensions. Amplitude and phase information between 1.2 µm and 1.7 µm were implemented as automatic differentiation-compatible interpolations.

Unlike the custom tapers in the interferometers, the directional couplers used in our networks are constructed to be all identical, with no trainable parameters. Due to the modular implementation of our design framework, it is possible to replace this specific directional coupler shown above with a different component, such as another directional coupler with a shorter or longer coupling length, one that uses bends with different geometries, or an entirely different coupler with a task-specific transfer function. For achieving smaller device footprints, other structures may be used in place of the directional couplers inside the interferometers. This is partly enabled by the design framework's ability to computationally separate the coupler response from that of the custom tapers, to achieve a modular simulation workflow. For instance, these couplers can be replaced with their more compact counterparts like those designed with inverse-design approaches [1, 2]. This can result in reducing the 4×30 µm^2 directional coupler footprint to typical inverse design device sizes below 4×4 µm^2 [3, 4]. The resulting network sizes can be improved by a factor of 4-5x, further increasing integration density.

II. INITIALIZATION AND OPTIMIZATION OF CUSTOM WAVEGUIDE TAPERS

The photonic network aims to optimize the geometry of custom waveguide tapers and the resulting $\theta(\lambda)$ according to the objective function specified. However, even though a representative neural network model can optimize for specific $\theta(\lambda)$, this mathematical procedure can easily yield final widths and lengths that are not appropriate for an actual photonic device or our specific network architecture. To this end, in order to design our photonic devices, we build our physics-informed network models with specific initialization considerations, numerous physical boundaries, and regularization schemes imposed on the trainable parameters, as detailed below.

A. Initialization and Constraints for Trainable Widths and Lengths

As our demonstrations are on the 220 nm SOI platform, the default waveguide width (w_{default}) for the entire network including input/output waveguides, directional couplers, and bends, is chosen to be 450 nm. As such, the final resulting widths in the custom tapers should also be relatively close to this default width, in order to ensure inherent fabrication compatibility of the designed devices, and also to minimize the excitation of higher-order modes as light propagates through these tapers.

Therefore, it is important that the array of custom waveguide widths are initialized relatively close to w_{default}, which we implement by $w_{\text{initial}} = w_{\text{default}} + p_w \cdot \Delta w + w_{\text{offset}}$ at the beginning of optimization. Here, p_w is a uniform random variable between -1 and 1, Δw is a user-chosen maximum deviation amplitude, and w_{offset} is a constant initial offset. The designer can choose to specify an initial offset, which is especially useful if the parameter updates exhibit monotonic tendencies during optimization, which can be a common occurrence in some types of artificial neural networks [5–7]. Throughout optimization and calculation of the corresponding transfer functions, we also clip the trainable widths between $w_{\text{min}} \leq w \leq w_{\text{max}}$, in order to ensure sufficient mode confinement within the waveguide core, and to limit the development of strongly guided higher-order modes in the waveguides. For the networks we demonstrated, these parameters were typically chosen to be 5 nm $\leq \Delta w \leq$ 20 nm, 30 nm $\leq w_{\text{offset}} \leq$ 50 nm, $w_{\text{min}} =$ 400 nm, and $w_{\text{max}} =$ 520 nm.

A similar implementation is used for the initialization of custom taper lengths with several minor differences. First, we initialize the lengths as $L_{\text{initial}} = L_{\text{max}} - p_L \cdot \Delta L$, where p_L is a random variable between 0 and 1, and ΔL is a user-chosen maximum deviation amplitude. We similarly clip the trainable lengths between $L_{\text{min}} \leq L \leq L_{\text{max}}$ throughout the optimization. Additionally, if the resulting taper length is shorter than L_{max}, in each iteration of the optimization procedure, we add a straight waveguide at the end of the taper with a length equal to $L_{\text{add}} = L_{\text{max}} - L$, ensuring that successive layers of MZIs align in the propagation direction. For demonstrated networks, these parameters were chosen to be $L_{\text{max}} =$ 10 µm and $L_{\text{min}} =$ 6 µm. With these parameter selections and 30 µm-long directional couplers, the resulting length of each MZI in our networks was 80 µm.

In addition to the initialization and imposed boundaries, the selection of the number of trainable widths per waveguide taper presents an important design consideration. By design, the ends of the custom tapers are fixed widths of w_{default}; and the trainable widths are placed with equal spacing in between. As such, the number of trainable widths (together with L_{min} and L_{max} parameters) directly influences the resulting taper angle, which can result in potential propagation losses and/or excitation of any higher-order modes if the taper has insufficient length

[8–10]. In our designs, we have chosen to place 5 uniformly-spaced trainable widths inside each waveguide taper, resulting in at least 1 µm spacing between them. Based on 3D-FDTD results, we find that a minimum taper length of $L_{\text{min}} = 1\ \mu\text{m} \times (\xi + 1)$ is sufficiently long for the low-loss operation of tapers between $w_{\text{min}} = 400$ nm and $w_{\text{max}} = 520$ nm, where ξ is the number of trainable widths in a single taper. The designer can also alternatively choose to place a greater number of trainable widths within each taper, and use a longer L_{min} parameter.

B. Regularizers

In addition to the initialization and constraints detailed above, two major regularization schemes are implemented in order to control the trainable widths through the iterative optimization. The first regularizer aims to reduce the difference between consecutive widths in a single waveguide taper, in order to prevent abrupt changes in width and potential resulting propagation losses. The second regularizer aims to limit the difference between any one of trainable widths and a reference width w_{ref} (which may be chosen to be the same as w_{default}), in order to maintain the optimized variables within the relative vicinity of this reference width. Both regularizers are calculated as L2-norms of the corresponding error vectors as

$$P_1 = \sum_{\text{all tapers}} \sum_{i=1}^{\xi-1} (w_i - w_{i+1})^2 \tag{1}$$

and

$$P_2 = \sum_{\text{all tapers}} \sum_{i=1}^{\xi} (w_i - w_{\text{ref}})^2 \tag{2}$$

where w_i are the individual trainable widths in each custom taper in a given photonic network. An overall regularization is computed as $P = \alpha_1 P_1 + \alpha_2 P_2$, where coefficients α_1 and α_2 are used to independently control the contribution strength of the two regularizers. The accumulated contribution of the regularizers is then implemented as an artificially-introduced loss at the end of the network, after which the overall objective function is calculated as

$$J(x) = \frac{1}{Q} \sum_{\lambda} \mid T_{\text{calculated}}(\lambda, x) e^{-P} - T_{\text{target}}(\lambda) \mid^2 \tag{3}$$

where Q is the number of wavelengths and x are design parameters including widths and lengths of the custom tapers. For the networks demonstrated here, we used $\alpha_1 = 3 \times 10^{-4}$ and $\alpha_2 = 1 \times 10^{-4}$. Depending on the photonic capabilities required, the designers may freely experiment with various contribution strengths and examine resulting taper profiles. As expected, stronger regularizations result in waveguides with more slowly varying widths, but also reduce the capability of the photonic network to replicate arbitrary transfer functions, due to the reduced effective degrees of freedom. Thanks to the computational efficiency of our network implementation, experimenting with different regularization contributions and comparing resulting taper structures is trivial from a designer's perspective.

III. HYPERPARAMETER CONSIDERATIONS FOR DEEP PHOTONIC NETWORKS

Similar to many other machine learning models, the selection of appropriate hyperparameters has key implications on the capability and scalability of our deep photonic networks, as well as the computational efficiency of their physics-informed, representative, artificial neural network models. In this section, we provide details on the effects of the number of interferometric layers and the number of trainable parameters per MZI on the final performance of our photonic networks.

A. Number of Interferometric Layers

The number of interferometric layers (the network depth) directly influences the number of trainable parameters, and is one of the key hyperparameters of our photonic networks. As demonstrated in Fig. 5, this network depth strongly affects the final performance and the tolerance against fabrication imperfections of the resulting photonic device. In Supp. Fig. 2, we perform similar investigations for the 75/25 power splitter and the spectral duplexer devices by comparing the final performance and robustness of 10 different devices designed from $M = 2$ to $M = 60$ layers, with the resulting number of trainable parameters ranging between 48 and 1440.

In Supp. Fig. 2(a) and (c), we plot the calculated errors for each type of photonic device and observe an initial decrease in this error with the increasing number of layers due to increased network capability. Minimum errors are reached around $M = 3$ to 5 layers for the 75/25 splitter, and with $M = 9$ to 14 layers for the duplexer. As the duplexer performs a more complicated spectral functionality, it requires a deeper, and therefore more capable network, with greater degrees of freedom. However, deeper networks also suffer more strongly from fabrication imperfections resulting from phase errors, and other propagation losses including those in directional couplers and S-bends. This can be verified from the general profile of the errors as a function of over-etch/under-etch offset Δw plotted in Supp. Fig. 2(b) and (d), where shorter networks generally demonstrate better (flatter) performance under strong etch offsets reaching 20 nm. Specifically for the 75/25 splitter, while the minimum error is reached with a 4-layer network, this 4-layer network exhibits significantly worse fabrication tolerance under 20 nm etch offsets, in comparison with the 3-layer network. Likewise, for the spectral duplexer, while the minimum error is reached with a 10-layer network, the fabrication tolerance of this 10-layer network is significantly inferior in comparison to the 6-layer and 7-layer networks. In comparing our devices, we note that the duplexer demonstrates better fabrication tolerance in these simulations than both of the power splitters. This difference can be attributed to the final error of the duplexer (2×10^{-4}) already being 1-2 orders of magnitude greater than the final errors for the power splitters (between 7×10^{-6} and 2×10^{-5}).

In general and also for our specific designs here, we consider both the final error and the fabrication tolerance of the resulting photonic networks. In cases where devices with different numbers of layers exhibit closely similar performances in calculated errors, we choose the device with a smaller number of layers in order to ensure a greater degree of robustness against fabrication variations, and also for compact device footprints.

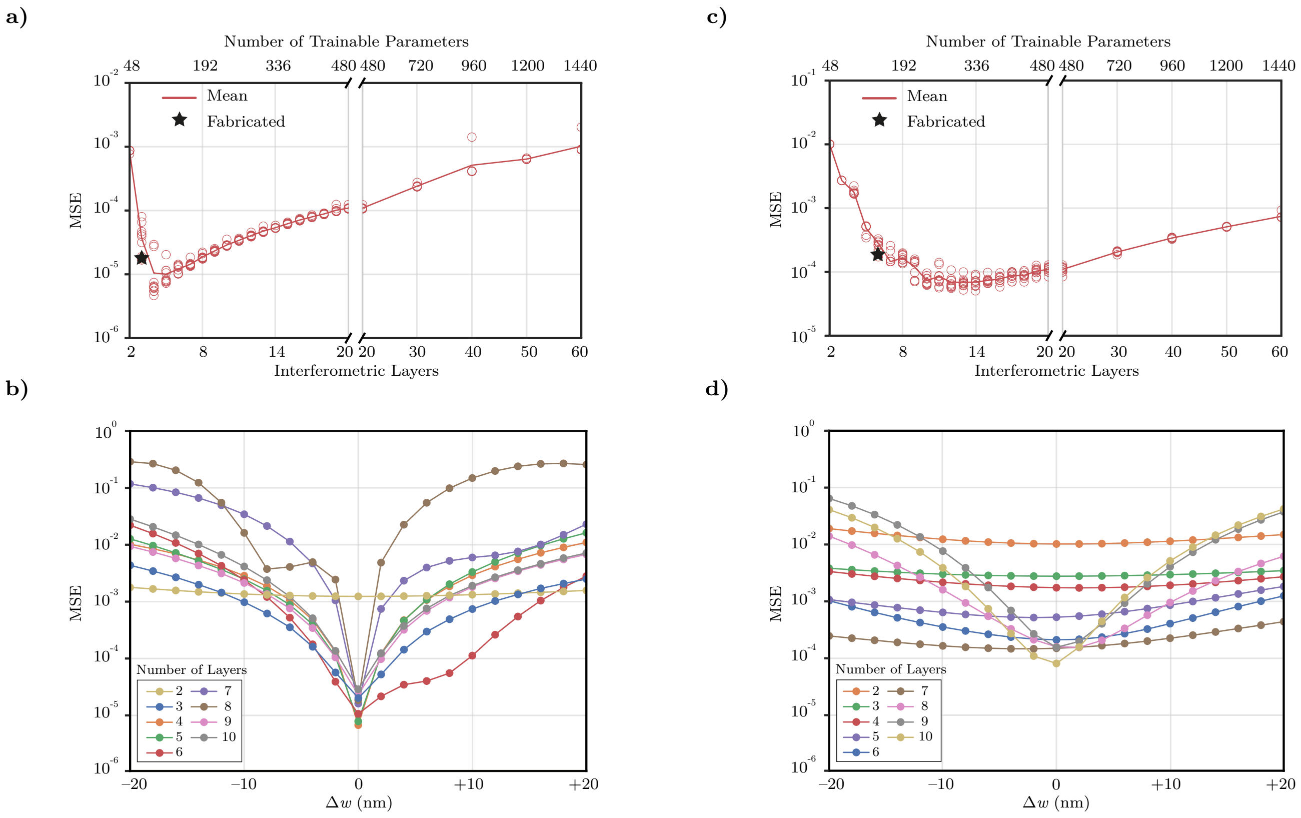

Supplementary Figure 2. Influence of network size on final device performance. Mean squared error for **(a)** 75/25 power splitter, and **(c)** spectral duplexer deep photonic networks as functions of the number of interferometric layers. The plotted error includes propagation loss in the directional couplers and the S-bends extracted from their 3D-FDTD simulations. For each network size, ten different randomly-initialized devices are optimized and depicted with red circles. Robustness against fabrication variations for **(b)** 75/25 power splitter, and **(d)** spectral duplexer. A greater number of interferometric layers initially reduces the calculated errors. However, longer devices suffer from stronger deviations from target functionality under fabrication variations.

B. Number of Trainable Parameters

The trainable parameters consist of the widths and the length for each custom taper in our photonic networks, as illustrated in Fig. 2. Specifically, each MZI in the network is made from four waveguide tapers built from trainable widths, four trainable lengths (one for each taper), and two fixed (non-trainable) directional couplers. By design, the network depth directly determines the number of trainable lengths. However, the number of trainable widths per custom taper is a designer-specified parameter. In the presented devices, we used five trainable widths for each taper, yielding a total of $4 \times (5 + 1) = 24$ trainable parameters per MZI. For each taper, these constituent widths and the taper length are used to calculate the accumulated phase, as shown in Fig. 1. As discussed in Supplementary Section 2A, we generally limit the number of trainable widths as $\xi \leq (L_{\text{min}} - 1\ \mu\text{m})/1\ \mu\text{m}$, in order to ensure slowly-varying waveguide geometries in the propagation direction for minimizing unwanted loss. For the 10 µm maximum taper lengths in our devices, this results in the recommended number of trainable widths being less than or equal to 9. However, within this bound, the number of trainable widths still remains a user-specifiable design choice. For our devices demonstrated here, we used $\xi = 5$ trainable widths per taper, as we find that there is a negligible added benefit beyond five trainable widths, since the required optimized phase profiles can already be reached in the custom

tapers. Additionally, the number of trainable widths $\xi > 5$ may also result in slightly longer optimization times, with no improvements in device performance.

IV. COMPUTATIONAL PERFORMANCE AND TIME REQUIREMENTS FOR NETWORK OPTIMIZATION

One of the most important advantages of our deep photonic network design framework is its ability to replace resource-extensive and time-consuming 3D-FDTD simulations with physically accurate, yet computationally efficient scattering matrix calculations. These computational requirements for underlying simulations are especially critical in design tasks where device parameters are repeatedly modified, and the device response is simulated in an iterative manner. Our design framework achieves computationally efficient and highly scalable operation through its use of state-of-the-art machine learning software and hardware infrastructure.

In Supp. Fig. 3, we demonstrate this computational performance for the design of several power splitters with various numbers and configurations of output ports, using a single Tesla V100 GPU. Specifically, we consider 2-port, 4-port, and 8-port broadband power splitters in evenly distributed and randomly distributed output power configurations, optimized at 32 evenly-spaced wavelengths between 1400-1600 nm. In Supp. Fig. 3(a), we plot the total time required for the convergence of each device as a function of the network depth. A relative convergence criterion of

$$\frac{\mid J(x)_{\text{current}} - J(x)_{\text{previous}} \mid}{\max(J(x)_{\text{current}}, J(x)_{\text{previous}})} < 10^{-3} \tag{4}$$

was used for all devices where $J(x)$ was defined in Eq.(3) above. As expected, the total optimization time scales with the number of interferometric layers (M) and the number of network outputs (N). As a function of these two parameters and the number of trainable widths per custom waveguide taper, the total number of trainable network parameters is given by

$$\begin{cases} 4(\xi+1)M, & N = 2 \\ 4(\xi+1)(\lceil \frac{M}{2} \rceil \lfloor \frac{N}{2} \rfloor + \lfloor \frac{M}{2} \rfloor \lfloor \frac{N-1}{2} \rfloor), & N \geq 3. \end{cases} \tag{5}$$

However, even for photonic networks as deep as 10 layers (800 µm device length), with 8 separate output ports, and a highly complicated photonic functionality like random and broadband output power splitting, the entire optimization procedure is completed in less than 12 minutes. This result presents superior scalability and multiple orders of magnitude advancements over optimization procedures based on 3D-FDTD simulations [11–15]. For smaller photonic networks with simpler functionalities, our optimizations complete in less than approximately 1 minute for networks of up to four layers. This capability of designing devices with physically accurate simulations within several minutes gives photonic designers the crucial ability to easily iterate through different versions of their photonic networks, and investigate device performance using different hyperparameters in their designs. The scalability of our design framework is also demonstrated by the per-iteration-time for each network optimization plotted in Supp. Fig. 3(b). Here, an iteration is defined as the computational operation of updating the network parameters once, after calculating

$J(x)$ and the required gradient $\nabla_x J$ using all 32 wavelengths in a parallelized manner. As a function of the network depth (and therefore the number of trainable parameters), the time required for each iteration on average ranges between a few milliseconds and a few tens of milliseconds. In addition to the modularity of our photonic network construction, this performance was also partly enabled by the use open-source deep learning software libraries JAX [16] and Trax [17]. These libraries allow automatic differentiation-compatible computations to be performed by CPU, GPU, or TPU accelerator hardware in a parallelized manner through just-in-time (JIT) compilation [18] and execution of required function calls. For our photonic networks, after a model is created, a certain preparation time is necessary in order for JIT compilation to complete, before iterative optimization can begin. For specific optimizations shown in Fig S3, this preparation time ranged between 8 and 650 seconds for 1-layer to 10-layer deep networks. This is included in the total optimization durations plotted in Supp. Fig. 3(a), but excluded from the per-iteration time plotted in Supp. Fig. 3(b).

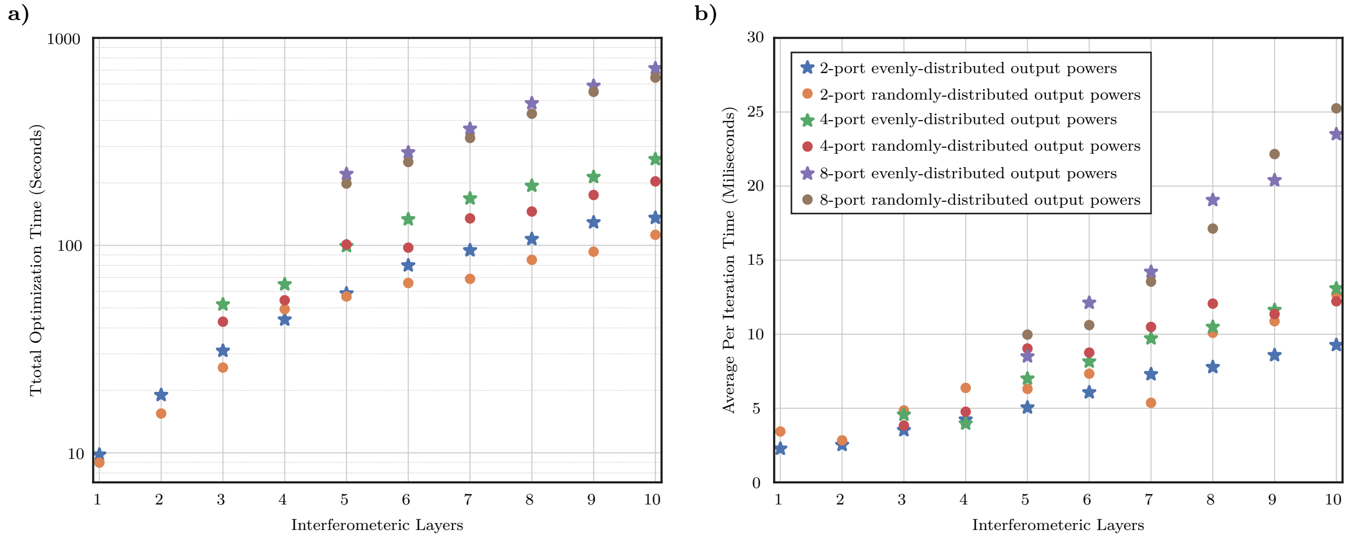

Supplementary Figure 3. Computational performance of the design framework for deep photonic networks as arbitrary power splitters. **(a)** Total optimization time for networks with different number of interferometric layers. **(b)** Average time per optimization iteration. Networks up to 10-layers deep with the number of output ports $N = 2$, 4, and 8 are optimized for broadband power splitting using 32 wavelengths between 1400 nm and 1600 nm. Separate photonic networks are designed with evenly distributed output powers, and randomly distributed output powers. Light is injected at the input number $\lfloor \frac{N}{2} \rfloor$ for all devices. All optimizations were performed using an open-source, end-to-end deep learning software library [16, 17], adaptive moment estimation optimizer [19], and a single Tesla V100 GPU. Optimizations were completed in several hundred iterations for each device, using a convergence criterion of 10^{-3} relative change in the overall objective $J(x)$.

For multi-objective optimizations, such as the example shown in Fig. 6, the specified objectives are all simulated simultaneously, where all physical simulations at different wavelengths, using different inputs, and for different etch-offsets are processed in parallel. This computational efficiency allows the optimization framework to scale well for the design of fabrication tolerant devices, and devices with target transfer functions across a large number of input-output pairs. As expected, the fabrication-tolerant optimization in Fig. 6(c) exhibits increased complexity due to the consideration of multiple objectives, resulting in a higher number of iterations needed for convergence in comparison to optimization under ideal conditions shown in Fig. 6(b). However, despite the doubling of the number of iterations from 1000 to 2000, we note that the total optimization time increased only by less than 5 seconds (from 45.5 s to 49.4 s), underscoring the efficiency of our framework. This can also be attributed to the initial part of the optimization

workflow used for JIT compilation of required mathematical operations, which remains independent of the number of iterations. As such, each additional iteration taking a few milliseconds adds minimal computational time to the overall optimization process.

In Supp. Fig. 3, indicated by "8-port randomly-distributed output powers" is a photonic network structurally consisting of 8 inputs and 8 outputs, for which a randomly specified, complex set of output power distributions (for one of the inputs) has been optimized for a total of 32 wavelengths between 1400 nm and 1600 nm. At a depth of 10 layers, this network is constructed from 35 unique MZIs in a cascaded geometry, resulting in a total of 840 trainable widths and lengths, achieving the same convergence criteria as all of our other demonstrations. For completeness of these results, we plot the simulated transmissions at each output port, together with a schematic diagram of this 8-port power splitter in Supp. Fig. 4. The target outputs consist of eight different and randomly-selected transmissions indicated by the dashed lines. The simulated final output transmissions as a function of wavelength are plotted with the solid curves for each one of the eight output ports. The results demonstrate good agreement between the target and simulated transmissions, indicating that the design capability of our design framework scales well to both wider and deeper photonic network structures. Moreover, achieving the desired photonic functionality at these scales does not sacrifice computational efficiency, as the entire optimization for this network only takes approximately 12 minutes.

V. CHARACTERIZATION OF REFLECTION IN DEEP PHOTONIC NETWORKS

We start by examining reflection in our interferometers through 3D-FDTD simulations shown in Supp. Fig. 5 from our 50/50 power splitter, and one of its optimized tapers. In simulation, the entire network's response in Supp. Fig. 5(a) indicates a total reflection of less than -50 dB throughout its entire bandwidth. In Supp. Fig. 5(b), we plot the reflection from just a single optimized taper (as shown in Fig. 1(d)), but make sure to select the taper with the widest width variations along its length to observe the worst possible reflections. For this single taper, the simulated reflections remain below -60 dB, indicating no practical influence of input-to-input "scattering" effects on the performance of demonstrated deep photonic networks. It is important to note that the width regularizers explained in Supplementary Section 2 play an important role in limiting the deviation of widths in a given taper, which also effectively minimize reflections and other scattering losses.

In addition to the simulations above, we also conducted experimental measurements of reflection, using the same 50/50 splitter network as an example. We first measure the reflection from our deep photonic network (dpn) with input and output grating couplers (gc) using a fiber circulator, yielding the combined reflection spectrum of the "gc + dpn + gc" structure. From this measurement, we extract the reflection spectrum corresponding only to the photonic network, through the following back-calculation procedure involving the measured reflection and transmission spectra of the "gc + gc" grating coupler input-output test structure on the same chip. The reflection-included scattering matrix for a grating coupler can be expressed as

$$S_{\text{gc+gc}} = \begin{bmatrix} t_{\text{gc}} & -jr_{\text{gc}} \\ -jr_{\text{gc}} & t_{\text{gc}} \end{bmatrix} \tag{6}$$

where t_{gc} and r_{gc} are the transmission and reflection amplitude coefficients of the grating coupler, respectively. The

combined scattering matrix for the "gc + gc" grating coupler test structure is calculated by the product of the corresponding transfer matrices, and is given by

$$S_{\text{gc+gc}} = TS(ST(S_{\text{gc}}) \times ST(S_{\text{gc}})) = \frac{1}{1+r_{\text{gc}}^2}\begin{bmatrix} t_{\text{gc}}^2 & -jr_{\text{gc}}(1+r_{\text{gc}}+r_{\text{gc}}^2) \\ -jr_{gc}(1+r_{gc}+r_{gc}^2) & t_{gc}^2 \end{bmatrix} \tag{7}$$

where we defined $ST()$ and $TS()$ functions to convert between scattering and transfer matrices for convenience [20]. Using this description, and the experimental results of transmission and reflection from our grating coupler test structure, we extract t_{gc} and r_{gc} parameters as a function of wavelength. Through a similar procedure, we then express the scattering matrix for the measured "gc + dpn + gc" device as

$$S_{\text{gc+dpn+gc}} = TS(ST(S_{\text{gc}}) \times ST(S_{\text{dpn}}) \times ST(S_{\text{gc}})) \tag{8}$$

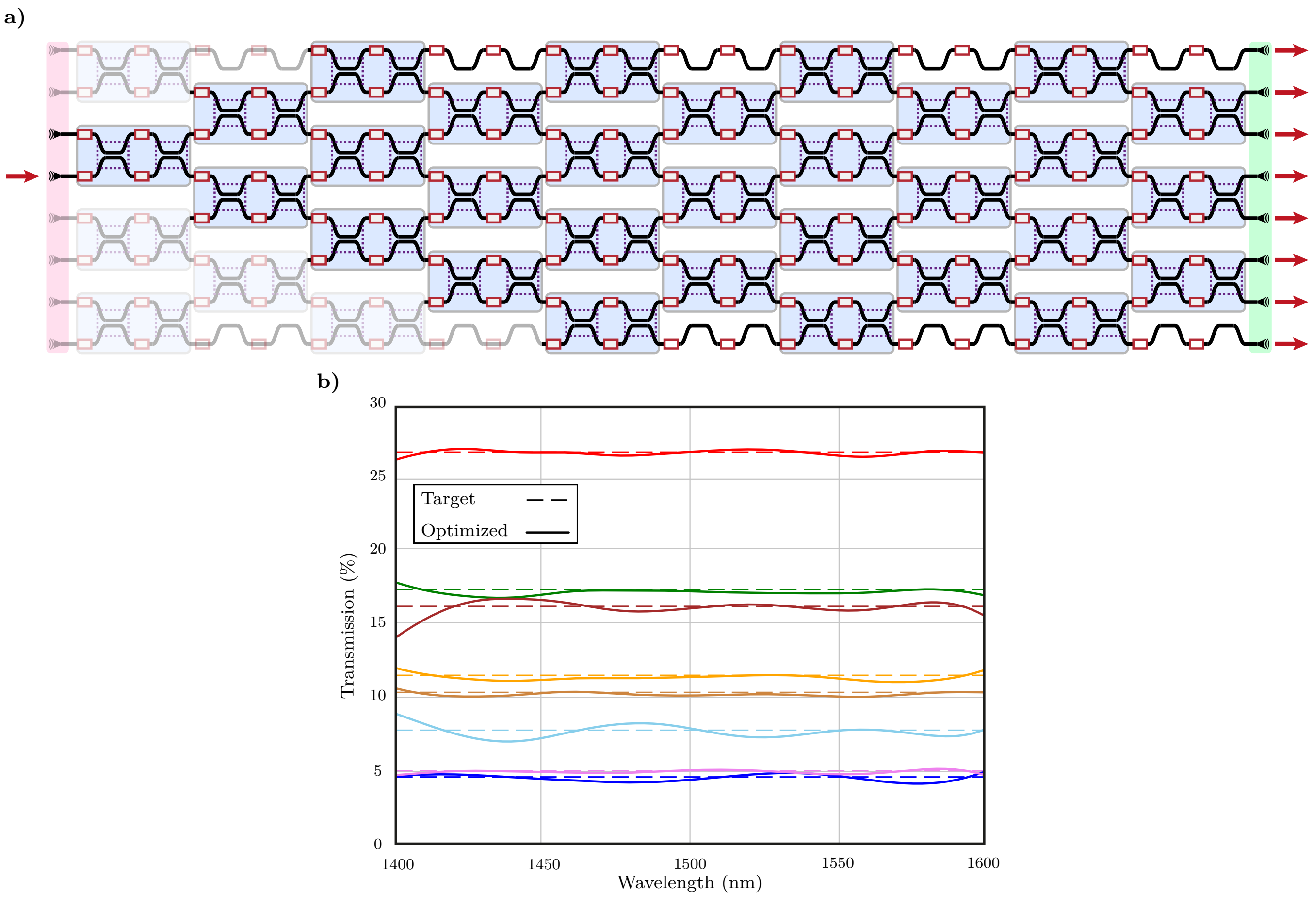

Supplementary Figure 4. 8-port deep photonic network with 10 layers, optimized for randomly-selected output powers. (a) Structure of the deep photonic network with a total of 840 trainable width and length parameters. Light injected at input number 4 propagates through 30 of the 35 placed MZIs. **(b)** Transmission response of eight randomly-selected, broadband transmission objectives specified at 32 evenly spaced wavelengths between 1400 nm and 1600 nm, as plotted by the horizontal dashed lines. The simulated final output transmissions are plotted by the solid curves for each output port.

where

$$S_{\mathrm{dpn}} = \begin{bmatrix} t_{\mathrm{dpn}} & -jr_{\mathrm{dpn}} \\ -jr_{\mathrm{dpn}} & t_{\mathrm{dpn}} \end{bmatrix} \tag{9}$$

represents the reflection-included scattering matrix for the input and one of the outputs of the deep photonic network, with corresponding transmission (t_{dpn}) and reflection (r_{dpn}) amplitude coefficients. This expression yields

$$S_{\mathrm{gc+dpn+gc}} = \frac{1}{1 + 2r_{\mathrm{gc}}r_{\mathrm{dpn}} + r_{\mathrm{gc}}^2(r_{\mathrm{dpn}}^2 + t_{\mathrm{dpn}}^2)} \begin{bmatrix} A & B \\ B & A \end{bmatrix}, \tag{10}$$

where

$$A = t_{\mathrm{gc}}^2 t_{\mathrm{dpn}}, \tag{11}$$

and

$$B = -j\left[2r_{\mathrm{gc}}^2 r_{\mathrm{dpn}} + r_{\mathrm{dpn}}t_{\mathrm{gc}}^2 + r_{\mathrm{gc}}^3(r_{\mathrm{dpn}}^2 + t_{\mathrm{dpn}}^2) + r_{\mathrm{gc}}(1 + r_{\mathrm{dpn}}^2 t_{\mathrm{gc}}^2 + t_{\mathrm{dpn}}^2 t_{\mathrm{gc}}^2)\right], \tag{12}$$

which is then used to back-calculate t_{dpn} and r_{dpn} parameters as a function of wavelength, using the previously extracted t_{gc} and r_{gc} from above. The results are plotted in Supp. Fig. 6 showing the experimentally obtained reflection and transmission spectra from the 50/50 splitter deep photonic network. The measured reflection from the entire deep photonic network remains below a maximum of -16.6 dB, and around -30 dB for the majority of the spectral range as shown in Supp. Fig. 6(a). Similarly, the transmission result now recalculated by taking reflections into account in Supp. Fig. 6(b) closely resembles the directly-measured transmission in Fig. 4(a), as also replotted in Supp. Fig. 6(b) for reference. These results verify practically negligible impacts of reflection from our deep photonic networks and their constituent components on the network performance.

VI. BAND-PASS FILTERS WITH DIFFERENT BANDWIDTHS

Our deep photonic network design infrastructure is also capable of arranging transmission objectives in order to create band-pass filters with high extinction-ratios and sharp spectral features. In order to achieve these metrics that

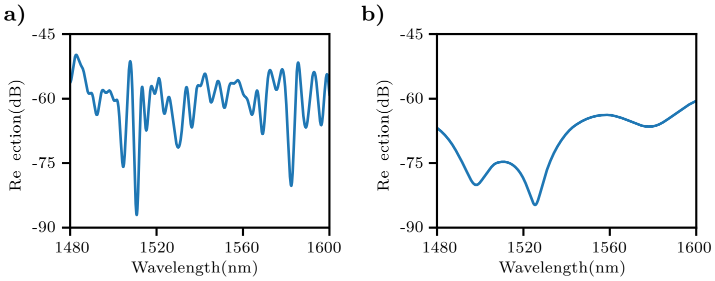

Supplementary Figure 5. 3D-FDTD simulation results of reflection in deep photonic networks. (a) Simulated reflection measured at the input of the 50-50 power splitter network. **(b)** Simulated reflection for one of the optimized tapers in the same photonic network.

are typically required in communications applications, we expand the deep photonic network structure with longer delays in the form of spirals as shown in Supp. Fig. 7(a). This structure enables band-pass optical transfer functions that are otherwise typically achieved with combinations of ring/disk resonators and/or MZIs [21–26]. The updated simulation of optical response for such a network also includes modular integration of lengths of these spiral waveguides as trainable parameters alongside the existing widths and lengths for the custom tapers. The phase acquired through this waveguide is a function of its trainable length, as illustrated in Supp. Fig. 7(b).

For this specific application, each network consists of a 1-input 2-output configuration with the labeled through and drop port outputs. Using this structure, we have designed three separate optical band-pass filters at a center wavelength of 1550 nm, with target bandwidths of 200 GHz, 100 GHz, and 50 GHz, whose simulated responses are shown in Supp. Fig. 7(c)-(e), respectively. The target bandwidth for each filter was controlled by specifying a range of wavelengths at which the entire optical input was transmitted to the drop port around the center wavelength. Inherently, achieving narrower filter bandwidths while maintaining a good extinction ratio represents a more difficult problem, and therefore, requires greater degrees of optical design freedom. Consequently, 6-layer, 9-layer, and 10-layer networks were used for designing the three filters, in decreasing order of optical bandwidth. The demonstrated bandwidths for the optimized devices were 201.90 GHz, 96.09 GHz, and 50.51 GHz for the three filters shown. At the center wavelength of filter transmission, extinction ratios of -48 dB, -25 dB, and -35 dB were obtained. As indicated by these transmission results, all three filters demonstrate agreement with their target specifications and achieve sufficiently high extinction ratios for communication applications. As an added capability for these applications, we have included a dB-based specification of optical transmission between the through and drop ports in our band-pass filter demonstrations. From an implementation perspective, this allows the optimizer to target a given maximum cross-talk between the outputs, and modify trainable width and length parameters towards achieving this goal. With this specification, our filters have achieved better than -20 dB cross-talk between the two outputs for the majority of design spectrum. Before optimization, spiral waveguide lengths were randomly initialized using $L_\mathrm{s} = L_\mathrm{default} - p_L \cdot \Delta L$, where p_L is a random variable between 0 and 1, and ΔL is a user chosen maximum deviation amplitude from the default, similar to the initialization of the lengths and widths of the custom tapers. After a set of optimization trials with different initializations, as is commonplace in machine learning model training [5–7], in order to achieve gradually narrower filter bandwidths, L_default of 50 µm, 80 µm, and 155 µm were used, respectively for the three devices. As anticipated, longer spiral lengths yielded photonic networks that are more suitable as narrow-bandwidth filters in our optimizations. However, the exact dependence of filter bandwidth on waveguide lengths remains a more complex

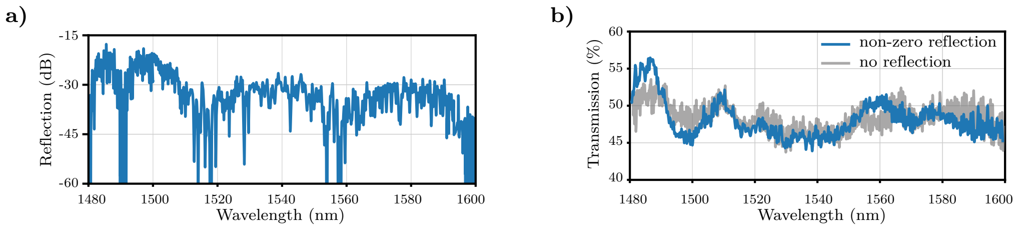

Supplementary Figure 6. Experimentally measured reflection and transmission results from the 50/50 splitter deep photonic network. **(a)** Reflection spectrum measured at the input of the network. **(b)** Transmission spectra at one of the outputs of the network, calculated by taking potential non-zero reflections into account (blue), and its comparison with the direct measurement assuming no reflections (gray).

function of optical power transmitted through each arm, which is influenced by the phase relationships between all custom tapers and spirals. The maximum final optimized spiral waveguide lengths remained below 55 µm for the 200 GHz filter, and 160 µm for all filters designed. Due to the compact structure of the spiral geometry, their placement can be planned in rectangular blocks adjacent to one of the interferometer arms as shown, with only a minor impact on the overall device footprint.

VII. DEVICES WITH OPTIMIZED DISPERSION PROFILES

Traditionally, on-chip constant dispersion is achieved using components like cascaded ring resonators [27–29] or Bragg gratings [30–32] due to highly linear group delay within their transmission/reflection bandwidths. At the wavelengths within their acceptable dispersion compensating bandwidth, these devices can inherently achieve near unity transmission, by the nature of their operation principles. A similar functionality can be accomplished by deep photonic networks; but this requires formulation of a combined objective with separately specified transmission and dispersion targets, in a fashion similar to the multi-objective designs we demonstrated. To do so, we separate the complex output of a photonic network into its amplitude and phase components, enabling calculation of transmission and group delay (as well as dispersion) results at each output port. For calculation of dispersion, the first and second derivatives of phase recorded and unwrapped at respective output ports are computed, using twice differentiable interpolations of effective index and directional coupler response. Compared to our transmission-only objectives, here we use a narrower wavelength spacing of 0.1 nm in order to avoid potential undersampling issues during unwrapping

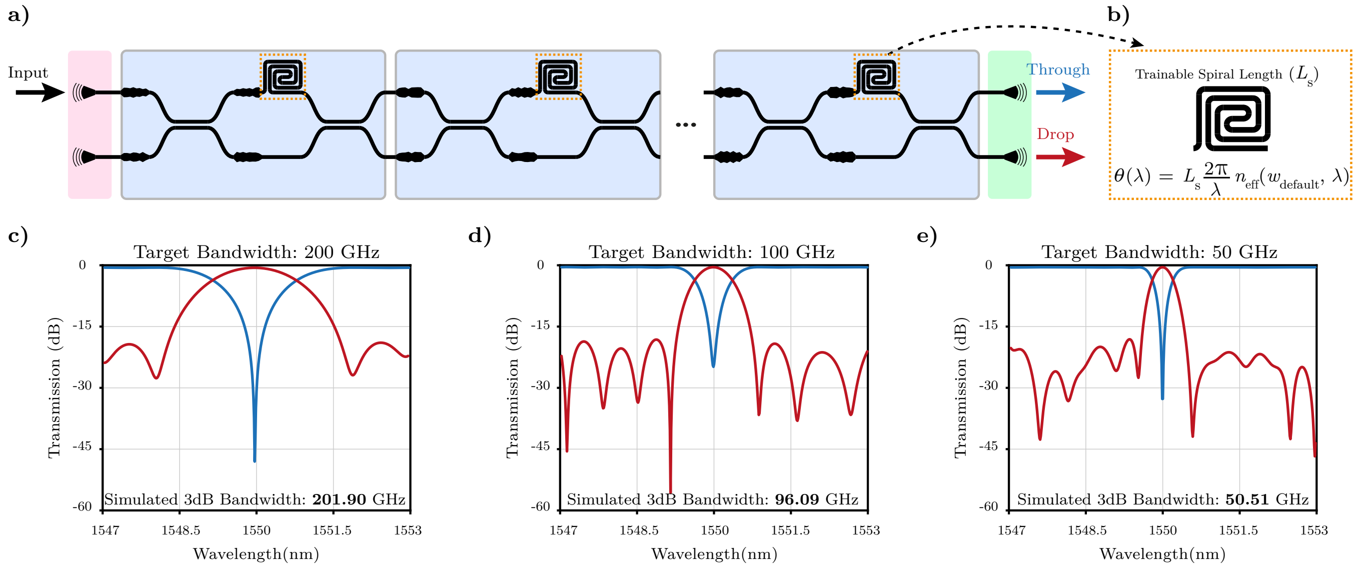

Supplementary Figure 7. Design of optical band-pass filters with the deep photonic network architecture. (a) Schematic drawing of the network structure where longer spiral waveguides have been added in each Mach-Zehnder interferometer for achieving band-pass functionality. **(b)** The phase accumulated in the spiral waveguide as a function of wavelength is calculated using its trainable length L_s and the default width of $w_{default} = 450$ nm. The transmission response at through and drop ports of **(c)** a 200 GHz band-pass filter with 6 layers of MZIs (150 trainable parameters), **(d)** a 100 GHz band-pass filter with 9 layers of MZIs (225 trainable parameters), and **(e)** a 50 GHz band-pass filter with 10 layers of MZIs (250 trainable parameters including trainable spiral waveguide lengths). The simulated final 3dB bandwidths are 201.90 GHz, 96.09 GHz, and 50.51 GHz for the three devices, respectively. Optimization for each one of the filters converges in several hundred iterations, in under three minutes of total computation time. All devices are optimized between 1547 nm and 1553 nm.

of this phase. We then define a figure of merit including all of these metrics to quantify the difference between target and calculated transmission and dispersion responses as

$$J(x) = \frac{1}{Q}\sum_{\lambda}\left((1-\eta)|T_{\text{calculated}}(\lambda, x) - T_{\text{target}}(\lambda)|^2 + \eta|D_{\text{calculated}}(\lambda, x) - D_{\text{target}}(\lambda)|^2\right) \tag{13}$$

where T and D indicate the transmission and dispersion responses of the network as a function of wavelength λ and trainable parameters x, and we defined an additional scaling parameter η. The control of this parameter η allows the designer to specify a relative weight between the transmission and dispersion objectives. We find experimenting with this parameter to be particularly useful in order to optimize for a certain amount of dispersion within the desired bandwidth, while still minimizing the power lost to other output ports to achieve low insertion loss (near unity transmission). For optimizing devices with this given figure of merit, we initialize and iteratively update trainable lengths and widths of a deep photonic network as before.

As proof of principle, we demonstrate two networks with a target constant dispersion of 0.5 ps/nm, with bandwidths of 3 nm and 6 nm, centered at 1550 nm, using 7 layers and 9 layers of MZIs, respectively. The geometry of these dispersion-compensating networks is structurally identical to the band-pass filters in Supp. Fig. 7(a); but the drop port remains unused as the entire output is collected from the through port in these devices. Here, the spiral waveguides were initialized to be around 47 µm in length, and then were iteratively optimized together with the custom tapers in the photonic network. The resulting group delays are plotted in Supp. Fig. 8(a) and S8(d), with both devices illustrating linear delay profiles confirming successful device optimizations. The dispersion is calculated from the derivative of this group delay, and plotted for the two devices in Supp. Fig. 8(b) and S8(e). The dispersion profiles deviate from the constant target of 0.5 ps/nm by 35-40 fs/nm towards the end of the specified bandwidth. Overall, the narrower-band device achieves better agreement with the dispersion target specified, due to the relative difficulty of achieving constant dispersion across wider optical bandwidths. A similar observation can be made regarding the transmission results in Supp. Fig. 8(c) and S8(f). The 9-layer device with 6 nm of target constant dispersion spectrum experiences slightly higher insertion loss, especially towards the end of the specified spectral range. As before, this emphasizes the relative difficulty of the target specification as simultaneously satisfying transmission and dispersion objectives across a wider bandwidth represents a more difficult optimization problem. Still, we observe better than -0.4 dB of insertion loss throughout the majority of the operation bandwidth, which is consistent with previous results from the literature [30, 33–35]. For compensating higher amounts of dispersion, multiple copies of the same networks can be cascaded in series, as their insertion loss remains relatively small. In comparison, while the demonstrated amounts of dispersion compensation in literature can exceed 10 ps/nm [30, 34–36], our network structure allows for not only constant anomalous dispersion for dispersion compensating applications, but also specification of arbitrary target dispersion profiles. These capabilities allow for unique design freedom in pulse-shaping [37–39] and quantum information processing applications [40], which typical dispersion compensators cannot provide. Even though cascaded ring resonators technically have the capability to achieve arbitrary dispersion profiles [27], the limitations become apparent when considering that each ring typically offers just two design parameters, thereby severely restricting the degrees of design freedom compared to our architecture.

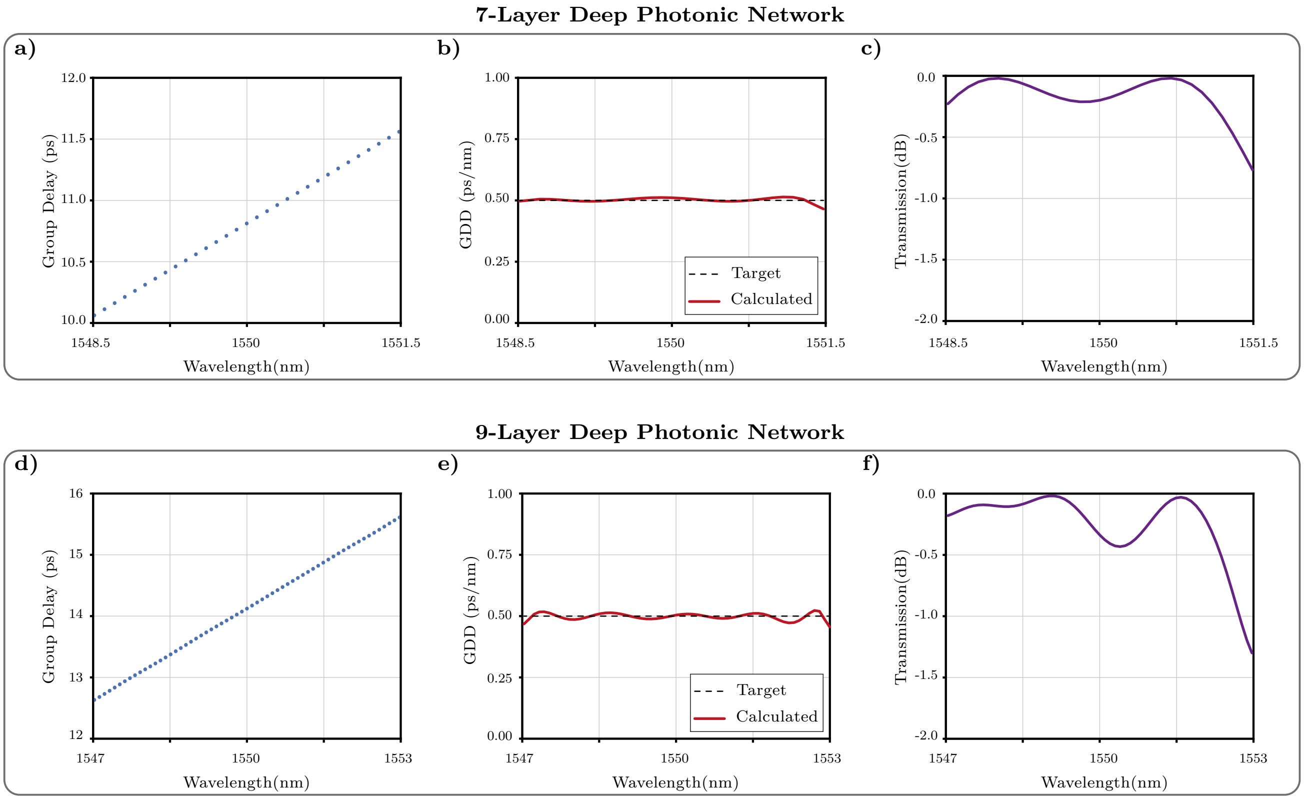

Supplementary Figure 8. Examples of deep photonic networks for dispersion optimization. Simulated group delay as a function of wavelength for **(a)** a 7-layer deep photonic network with 175 trainable parameters, and **(d)** a 9-layer deep photonic network with 225 trainable parameters (these parameters include the lengths and widths in custom tapers as well as the lengths of spiral waveguides). The network structure is identical to the one illustrated in Supp. Fig. 7(a); but the entire output is collected from the through port. **(b)**, **(e)** Spectral dispersion profiles of the two optimized devices. Both devices demonstrate near-constant dispersion throughout their optimized bandwidth of 3 nm and 6 nm, respectively. A target constant dispersion of 0.5 ps/nm was used. **(c)**, **(f)** Transmission through the devices remains better than -0.4 dB for the majority of their optimized spectrum. Some more insertion loss is observed towards the end of the specified spectral range. (GDD: group delay dispersion)

VIII. COMPARISON AND LIMITATIONS OF FUNCTIONAL NETWORK CAPABILITY

By the general nature of their design, MZI-based photonic networks are larger in size compared to devices obtained with free-form inverse-design techniques [1, 2]. Functionally similar beam splitting or spectral splitting capabilities have also been reported with such inverse design methods, resulting in footprints ranging from approximately 5 μm^2 to 100 μm^2 [11, 15, 41–44]. However, the diversity and bandwidths of optical capabilities achieved by these methods are limited to more basic operations across narrower bandwidths, due to the computational difficulty in addressing arbitrary design objectives requiring larger device sizes. Yet, even though our deep photonic networks demonstrate superior computational efficiency and higher design freedom, there are also practical limitations to their size and functionality, such as those influenced by propagation losses, accumulation of fabrication errors, and phase disorder due to sidewall roughness.

- Of these factors, we expect typical SOI waveguide propagation losses of 2-3 dB/cm not to practically introduce any additional design considerations specific to our photonic networks, since any propagation-related losses remain balanced between our symmetrically-placed interferometer arms by design. Using an MZI length of 80

µm, even for the longest, 10-layer device we have shown in Supp. Fig. 4, this corresponds to an expected total loss of approximately 3 dB/cm × 80 µm × 10 = 0.24 dB, which is acceptable for many on-chip optical applications. It is also important to note that the symmetric design of all interferometer arms in a given layer ensures propagation through an equal number of directional couplers and S-bends for each arm. The expected total loss through these individual components is already extracted from 3D-FDTD simulations, and is included in our analysis shown in Fig. 5 and Supp. Fig. 2. Up to a network depth of 10 layers, these plots illustrate that the optimized figures of merit remain below 10^{-4} (in line with the figures of merit obtained for the fabricated devices), indicating no practical influence of the added scattering losses due to the S-bends or directional couplers.

- Accumulation of fabrication errors through cascaded nodes presents an important challenge for any mesh-like structure. For deep photonic networks, since each individual device simulation can be performed on the order of milliseconds, our design framework enables building inherent tolerance to fabrication errors, as already shown in Fig. 6. Through this approach, the custom tapers are automatically optimized by taking into account multiple different under-etched and over-etched versions of the same device. We expect this added capability to provide significant flexibility in selecting the most appropriate designs for a specific task, which may also be influenced by fabrication methods used. However, as we have already discussed, longer devices naturally exhibit worse tolerances to fabrication errors accumulating through multiple interferometric layers (see Fig. 5(b), Supp. Fig. 2(b), and Supp. Fig. 2(d)). Therefore, for networks deeper than 10 layers, we expect practical limitations towards achieving similar levels of fabrication tolerance, which may require electrical, post-fabrication tuning.

- Longer devices also suffer from potential phase errors due to sidewall roughness in the fabricated waveguides. This is an especially well-known problem in high-index contrast platforms, as the larger index difference results in phase errors greater in magnitude. This is also an important reason behind the preference of certain optical phased array demonstrations toward SiN guiding platforms instead of Si, due to the comparatively lower index contrast and the smaller magnitude of resulting phase errors [45–48]. Using the 220 nm-thick commercially available SOI platform from IMEC [49], we have observed no detrimental effects on device performance due to this phenomenon for our experimentally demonstrated networks in Fig. 4, up to a network depth of 6 layers. In case of phase errors for deeper networks, a similar approach to implementing optical phase arrays in SiN platforms can be undertaken, since our design framework remains generalizable to other waveguide materials or platforms. While the diversity of optical operations and capabilities in design freedom we have shown can provide solutions for many optical applications within less than 10 layers, the presented design framework can be easily generalized to other guiding materials with smaller phase errors. It may also be possible to implement inherent fabrication tolerance to target these phase errors, in a similar fashion to achieving near-optimal optical responses for over-etch/under-etch scenarios discussed above.

SUPPLEMENTARY REFERENCES

[1] Molesky, S. *et al.* Inverse design in nanophotonics. *Nature Photonics* **12**, 659–670 (2018).

[2] Wiecha, P. R., Arbouet, A., Girard, C. & Muskens, O. L. Deep learning in nano-photonics: inverse design and beyond. *Photonics Research* **9**, B182–B200 (2021).

[3] Piggott, A. Y., Petykiewicz, J., Su, L. & Vučković, J. Fabrication-constrained nanophotonic inverse design. *Scientific reports* **7**, 1786 (2017).

[4] Piggott, A. Y. *et al.* Inverse-designed photonics for semiconductor foundries. *ACS Photonics* **7**, 569–575 (2020).

[5] Sutskever, I., Martens, J., Dahl, G. & Hinton, G. On the importance of initialization and momentum in deep learning. In *Proceedings of the 30th International Conference on Machine Learning*, no. 3 in Proceedings of Machine Learning Research, 1139–1147 (PMLR, 2013).

[6] Alom, M. Z. *et al.* A state-of-the-art survey on deep learning theory and architectures. *electronics* **8**, 292 (2019).

[7] Hanin, B. & Rolnick, D. How to start training: The effect of initialization and architecture. *Advances in Neural Information Processing Systems* **31** (2018).

[8] Milton, A. & Burns, W. Mode coupling in optical waveguide horns. *IEEE Journal of Quantum Electronics* **13**, 828–835 (1977).

[9] Fu, Y., Ye, T., Tang, W. & Chu, T. Efficient adiabatic silicon-on-insulator waveguide taper. *Photonics Research* **2**, A41–A44 (2014).

[10] Zou, J. *et al.* Short and efficient mode-size converter designed by segmented-stepwise method. *Optics letters* **39**, 6273–6276 (2014).

[11] Piggott, A. Y. *et al.* Inverse design and demonstration of a compact and broadband on-chip wavelength demultiplexer. *Nature Photonics* **9**, 374–377 (2015).

[12] Lu, J. & Vučković, J. Nanophotonic computational design. *Optics express* **21**, 13351–13367 (2013).

[13] Jia, H., Zhou, T., Fu, X., Ding, J. & Yang, L. Inverse-design and demonstration of ultracompact silicon meta-structure mode exchange device. *Acs Photonics* **5**, 1833–1838 (2018).

[14] Zhang, G. & Liboiron-Ladouceur, O. Scalable and low crosstalk silicon mode exchanger for mode division multiplexing system enabled by inverse design. *IEEE Photonics Journal* **13**, 1–13 (2021).

[15] Zhang, G., Xu, D.-X., Grinberg, Y. & Liboiron-Ladouceur, O. Experimental demonstration of robust nanophotonic devices optimized by topological inverse design with energy constraint. *Photonics Research* **10**, 1787–1802 (2022).

[16] Bradbury, J. *et al.* JAX: composable transformations of Python+NumPy programs, http://github.com/google/jax (2018). URL `http://github.com/google/jax`.

[17] Trax: an end-to-end library for deep learning that focuses on clear code and speed, https://github.com/google/trax (2020). URL `https://github.com/google/trax`.

[18] Aycock, J. A brief history of just-in-time. *ACM Computing Surveys (CSUR)* **35**, 97–113 (2003).

[19] Kingma, D. P. & Ba, J. Adam: A method for stochastic optimization. *arXiv preprint arXiv:1412.6980* (2014).

[20] Saleh, B. E. & Teich, M. C. *Fundamentals of photonics* (john Wiley & sons, 2019).

[21] Dai, T. *et al.* Bandwidth and wavelength tunable optical passband filter based on silicon multiple microring resonators. *Optics letters* **41**, 4807–4810 (2016).

[22] Zhang, B. *et al.* Compact multi-million q resonators and 100 mhz passband filter bank in a thick-soi photonics platform. *Optics Letters* **45**, 3005–3008 (2020).

[23] Eid, N. *et al.* Fsr-free silicon-on-insulator microring resonator based filter with bent contra-directional couplers. *Optics express* **24**, 29009–29021 (2016).

[24] Orlandi, P. *et al.* Reconfigurable silicon filter with continuous bandwidth tunability. *Optics letters* **37**, 3669–3671 (2012).

[25] Ding, Y. *et al.* Bandwidth and wavelength-tunable optical bandpass filter based on silicon microring-mzi structure. *Optics express* **19**, 6462–6470 (2011).

[26] Soltani, M., Yegnanarayanan, S. & Adibi, A. Ultra-high q planar silicon microdisk resonators for chip-scale silicon photonics.

Optics express **15**, 4694–4704 (2007).

[27] Madsen, C. K. *et al.* Integrated all-pass filters for tunable dispersion and dispersion slope compensation. *IEEE Photonics Technology Letters* **11**, 1623–1625 (1999).

[28] Sorianello, V. *et al.* 100gb/s polmux-nrz transmission at 1550nm over 30km single mode fiber enabled by a silicon photonics optical dispersion compensator. In *Optical Fiber Communication Conference*, W2A–31 (Optica Publishing Group, 2018).

[29] Takahashi, H., Inohara, R., Nishimura, K. & Usami, M. Expansion of bandwidth of tunable dispersion compensator based on ring resonators utilizing negative group delay. *Journal of lightwave technology* **24**, 2276 (2006).

[30] Sahin, E., Ooi, K., Png, C. & Tan, D. Large, scalable dispersion engineering using cladding-modulated bragg gratings on a silicon chip. *Applied Physics Letters* **110** (2017).

[31] Giuntoni, I. *et al.* Continuously tunable delay line based on soi tapered bragg gratings. *Optics express* **20**, 11241–11246 (2012).

[32] Liu, S., He, J. & Dai, D. Tunable dispersion compensator based on taper bragg gratings with heating-engineering. In *2021 Asia Communications and Photonics Conference (ACP)*, 1–3 (IEEE, 2021).

[33] Liu, S. *et al.* On-chip circulator-free chirped spiral multimode waveguide grating for dispersion management. *ACS Photonics* **10**, 1654–1661 (2023).

[34] Wang, X., Zhao, Y., Ding, Y., Xiao, S. & Dong, J. Tunable optical delay line based on integrated grating-assisted contradirectional couplers. *Photonics Research* **6**, 880–886 (2018).

[35] Yuan, W. & Dong, J. High linearity optical delay line based on cascaded multimode waveguide bragg gratings. In *Thirteenth International Conference on Information Optics and Photonics (CIOP 2022)*, vol. 12478, 570–576 (SPIE, 2022).

[36] Waqas, A., Melati, D. & Melloni, A. Cascaded mach–zehnder architectures for photonic integrated delay lines. *IEEE Photonics Technology Letters* **30**, 1830–1833 (2018).

[37] Khan, M. H. *et al.* Ultrabroad-bandwidth arbitrary radiofrequency waveform generation with a silicon photonic chip-based spectral shaper. *Nature Photonics* **4**, 117–122 (2010).

[38] Heck, M. J. *et al.* Design, fabrication and characterization of an inp-based tunable integrated optical pulse shaper. *IEEE journal of quantum electronics* **44**, 370–377 (2008).

[39] Weiner, A. M. Ultrafast optical pulse shaping: A tutorial review. *Optics Communications* **284**, 3669–3692 (2011).

[40] Notaros, J. *et al.* Programmable dispersion on a photonic integrated circuit for classical and quantum applications. *Optics express* **25**, 21275–21285 (2017).

[41] Ren, Y. *et al.* Genetic-algorithm-based deep neural networks for highly efficient photonic device design. *Photonics Research* **9**, B247–B252 (2021).

[42] Kim, J. *et al.* Experimental demonstration of inverse-designed silicon integrated photonic power splitters. *Nanophotonics* **11**, 4581–4590 (2022).

[43] Tahersima, M. H. *et al.* Deep neural network inverse design of integrated photonic power splitters. *Scientific reports* **9**, 1368 (2019).

[44] Ma, H., Huang, J., Zhang, K. & Yang, J. Inverse-designed arbitrary-input and ultra-compact 1× n power splitters based on high symmetric structure. *Scientific Reports* **10**, 11757 (2020).

[45] Poulton, C. V. *et al.* Large-scale silicon nitride nanophotonic phased arrays at infrared and visible wavelengths. *Optics letters* **42**, 21–24 (2017).

[46] Sun, C. *et al.* Large-scale and broadband silicon nitride optical phased arrays. *IEEE Journal of Selected Topics in Quantum Electronics* **28**, 1–10 (2022).

[47] Prost, M. *et al.* Optical phased array with on-chip phase calibration. *Optics Letters* **47**, 6081–6084 (2022).

[48] Dwivedi, S. *et al.* Calibration-free si-sin optical phased array. In *Integrated Photonics Research, Silicon and Nanophotonics*, IM4A–3 (Optica Publishing Group, 2019).

[49] Bogaerts, W. *et al.* Nanophotonic waveguides in silicon-on-insulator fabricated with cmos technology. *Journal of Lightwave Technology* **23**, 401 (2005).
